# Analysis of risk factors for Epstein–Barr virus reactivation and progression to post-transplant lymphoproliferative disorder in pediatric patients undergoing allogeneic hematopoietic stem cell transplantation

**DOI:** 10.3389/fped.2025.1627990

**Published:** 2025-07-17

**Authors:** Lanzhou Jia, Hui Yang, Ya Zhou, Yan Meng, Luying Zhang, Xiaoying Lei, Xianming Guan, Jie Yu, Ying Dou

**Affiliations:** ^1^ Department of Hematology and Oncology, Children’s Hospital of Chongqing Medical University, Chongqing, China; ^2^ National Clinical Research Center for Child Health and Disorders, Ministry of Education Key Laboratory of Child Development and Disorders, China International Science and Technology Cooperation Base of Child Development and Critical Disorders, Children’s Hospital of Chongqing Medical University, Chongqing, China; ^3^ Chongqing Key Laboratory of Child Infection and Immunity, Children’s Hospital of Chongqing Medical University, Chongqing, China

**Keywords:** allogeneic hematopoietic stem cell transplantation, pediatric, Epstein–Barr virus (EBV), reactivation, post-transplant lymphoproliferative disorder (PTLD)

## Abstract

**Background:**

Post-transplant infections are common complications, and the reasons are pre-transplant conditioning, time required for post-transplant immune reconstitution, and use of immunosuppressive agents. We aimed to analyze the risk factors for Epstein–Barr virus (EBV) reactivation and its progression to post-transplant lymphoproliferative disorder (PTLD) following allogeneic hematopoietic stem cell transplantation (allo-HSCT) in children and to determine the EBV PCR diagnostic threshold for PTLD.

**Methods:**

We retrospectively analyzed clinical data of 309 patients who underwent allo-HSCT without engraftment failure at the Children's Hospital of Chongqing Medical University from January 1, 2016, to December 21, 2021. The occurrences of EBV reactivation and PTLD were also recorded. The risk factors for EBV reactivation and progression to PTLD were analyzed, and the diagnostic threshold for PTLD was determined using whole-blood EBV PCR.

**Results:**

Among 309 pediatric patients, 256 experienced EBV reactivation within one year and 12 progressed to PTLD. Univariate and multivariate analyses indicated that ATG was the independent risk factor for EBV reactivation. Grade III-IV acute graft-vs.-host disease (aGVHD) was the risk factor for PTLD after EBV reactivation.

**Conclusions:**

Post-transplant EBV reactivation is a common complication after allo-HSCT, but rarely progresses to PTLD. Identification of the risk factors for PTLD and regular monitoring of the EBV-DNA load play important roles in prevention and cure of PTLD after HSCT.

## 1 Introduction

Hematopoietic stem cell transplantation (HSCT) is a therapeutic procedure involving the transplantation of hematopoietic stem cells, derived from the bone marrow, peripheral blood, or umbilical cord blood of donors, for partial or entire reconstruction of a recipient's hematopoietic and immune systems. It is an effective treatment for malignant and nonmalignant hematologic diseases. However, due to factors such as pre-transplant conditioning, the time required for post-transplant immune reconstitution, and the use of immunosuppressive agents, infections are common complications of transplantation. Epstein–Barr Virus (EBV), a DNA virus belonging to the γ-herpesvirus family, infects over 90% of the global population (1). After initial infection, EBV establishes a latent infection in memory B cells. In the context of post-transplant immunosuppression, EBV reactivation is a common cause of infection. Moreover, EBV reactivation is associated with various clinical diseases, ranging from simple fever to post-transplant lymphoproliferative disorder (PTLD), characterized by uncontrolled tumor-like proliferation of lymphoid or plasma cells and a high mortality rate (2). In recent decades, the incidence of PTLD has increased due to the growing use of HSCT, introduction of new immunosuppressive agents and treatment regimens, enhanced understanding of PTLD, and improved diagnostic accuracy (3).

The reported incidence of post-transplant EBV reactivation ranges from 13% to 82%, (4–10) and that of post-transplant PTLD varies from 0.79% to 14% (6, 11–16). Previous studies have identified risk factors for EBV reactivation, including use of anti-thymocyte immunoglobulin (ATG), cytomegalovirus viremia, pre-transplant EBV seropositivity, and different types of transplantation (14, 16, 17). Risk factors for PTLD after EBV reactivation include *T*-cell depletion (ATG and ex vivo *T*-cell depletion), human leukocyte antigen (HLA)-mismatched transplantation, umbilical cord blood stem cell transplantation, splenectomy before transplantation, severe acute or chronic graft-vs.-host disease, mesenchymal stem cell infusion, concurrent cytomegalovirus (CMV) infection, age, and underlying diseases (2, 3, 7, 11, 13, 18).

Rituximab, a monoclonal anti-CD20 antibody, is effective in treating EBV reactivation and in reducing the risk and mortality of PTLD (12, 13). However, its use may delay B-cell reconstitution and increase the risk of post-transplant infections, and few patients with EBV reactivation progress to PTLD. Therefore, appropriate selection of patients for rituximab treatment is crucial. The sixth European Conference on Infections in Leukemia (ECIL-6) guidelines (2) recommend regular monitoring of the EBV DNA load in transplant recipients.

In this study, we aimed to determine the incidence and outcomes of post-transplant EBV reactivation and PTLD at our center, identify the risk factors, and determine the whole-blood EBV viral load threshold for preemptive rituximab treatment.

## 2 Materials and methods

### 2.1 Study participants

This retrospective study included patients aged <18 years who underwent allogeneic hematopoietic stem cell transplantation (allo-HSCT) at the Children's Hospital of Chongqing Medical University between 2016 and 2021 and achieved successful engraftment.

### 2.2 Definitions and diagnostics

HLA matched was defined as 10/10 matched, HLA mismatched was defined as ≤9/10 matched; EBVemia and CMVemia was defined as whole-blood PCR exceeding 400 copies/ml, EBV reactivation was defined as two consecutive EBV-DNA above 1,000 copies/ml post-transplantation (19). The diagnosis of EBV-associated PTLD was defined as proven or probable according to the published definition. Probable PTLD was defined as significant lymphadenopathy, hepatosplenomegaly, or other end-organ manifestations accompanied by significant EBV DNAemia and the absence of other documented cause. Proven PTLD was defined as the detection of EBV in tissue specimen accompanied by symptoms and/or signs from the affected organ.

### 2.3 EBV prevention, monitoring, and treatment

Before transplantation, the patients underwent serological tests for EBV antibody screening and whole blood PCR, and antiviral prophylaxis with acyclovir was administered. Routine whole-blood EBV PCR monitoring began first week after transplantation and was continued weekly until discharge. Monitoring was performed biweekly from discharging to 100 days post-transplantation, followed by monthly monitoring for up to one year after transplantation. If the PCR load was observed to be significantly increased in two consecutive measurements or if a high PCR load was observed with fever, immunosuppressive agents were reduced proportionally. If the viral load did not decrease after immunosuppression reduction, and the whole blood EBV viral loads in two consecutive measurements exceeded 10^6^ copies/ml, preemptive rituximab treatment was considered. For patients clinically diagnosed with PTLD, rituximab was administered at 375 mg/m^2^ weekly for one to four weeks.

### 2.4 Statistical analysis

Data analysis was performed using SPSS [Computer Software]. Version 25.0. Armonk, NY: IBM Corp; 2017. The cumulative incidence of EBV infection was determined using the Kaplan–Meier method. Categorical data are presented as counts (percentages). Group comparisons were performed using the chi-squared test or Fisher's exact test. Nonparametric tests were conducted for between-group comparisons of continuous data. Variables with *P* values <0.05 in univariate analysis were included in the multivariate analysis, and logistic regression was employed to identify independent risk factors for EBV reactivation and progression to PTLD. Statistical significance was set at *P* < 0.05. Receiver operating characteristic (ROC) curves were used to determine the optimal cutoff point for EBV load for diagnosing PTLD.

## 3 Results

### 3.1 Patient and transplantation characteristics

A total of 309 pediatric patients were included in the study, with a median age of three years (range, 0.3–14.8 years). The clinical characteristics of the 309 patients are summarized in Table 1. All patients receive a prophylactic regimen for GVHD based on cyclosporine, in combination with Mycophenolate Mofetil and/or methotrexate.

**Table 1:** Characteristics of allogeneic hematopoietic stem cell transplantation (allo-HSCT).

Parameter	Total, *n* (%)
Sex	
Men	218 (70.6)
Women	91 (29.4)
Donor type	
Unrelated	210 (68.0)
Sibling	69 (22.3)
Related (non-sibling)	30 (9.7)
HLA-matching	
Matched	164 (53.1)
Mismatched	145 (46.9)
Stem cell source	
Peripheral blood	289 (93.5)
Bone marrow	5 (1.6)
Umbilical cord blood	8 (2.6)
Peripheral blood + Umbilical cord blood	1 (0.3)
Bone marrow + Umbilical cord blood	5 (1.6)
Peripheral blood + Bone marrow	1 (0.3)
Diagnosis	
Thalassemia	101 (32.7)
Primary immunodeficiency disease (PID)	138 (44.7)
WAS	45 (14.6)
CGD	12 (3.9)
HIGM	14 (4.5)
XLP	35 (11.3)
XLA	5 (1.6)
X-SCID	7 (2.3)
CID	6 (1.9)
CN	6 (1.9)
Other PID	8 (2.6)
Leukemia	43 (13.9)
Aplastic anemia	22 (7.1)
Other disease^a^	5 (1.6)
Use of ATG during conditioning	
Yes	257 (83.2)
No	52 (16.8)
EBV PCR before transplantation	
Positive	94 (30.4)
Negative	215 (69.6)
CMV PCR before transplantation	
Positive	28 (9.1)
Negative	281 (90.9)
EBV serostatus of recipient before transplantation	
IgG+	247 (79.9)
IgG±	14 (4.5)
IgG−	48 (15.5)

EBV PCR-positive: whole blood EBV DNA load >400 copies/ml; CMV PCR-positive: whole blood CMV DNA load >400 copies/ml.

HLA, human leukocyte antigen; ATG, anti-thymocyte immunoglobulin; EBV, Epstein–Barr virus; CMV, cytomegalovirus; WAS, Wiskott-Aldrich syndrome; CGD, chronic granulomatous disease; HIGM, hyper-immunoglobulin M syndromes; XLP, X-linked lymphoproliferative disease; XLA, X-linked agammaglobulinemia; X-SCID, X-linked severe combined immunodeficiency; CID, combined immunodeficiency; CN, congenital neutropenia; IgG, immunoglobulin G.

^a^ Other diseases included two cases of propionic acidemia, one case of hemophagocytic lymphohistiocytosis, one case of myelodysplastic syndrome, and one case of myeloid sarcoma.

### 3.2 EBV reactivation after transplantation

**Figure 1:**
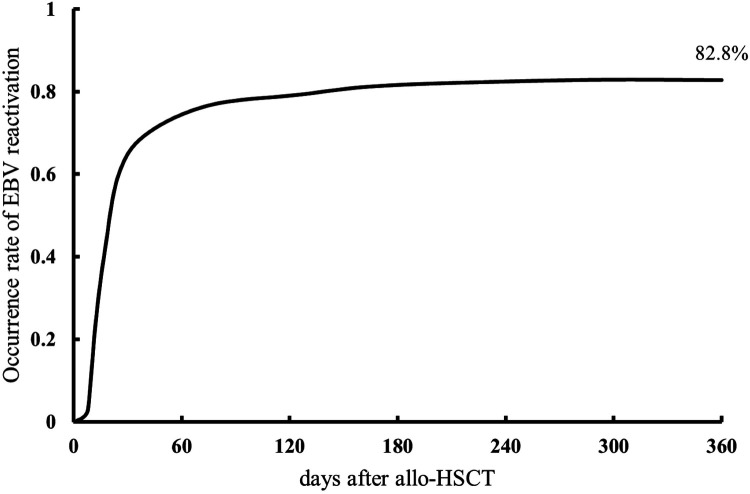
Cumulative incidence of Epstein–Barr virus (EBV) reactivation within one year of allogeneic hematopoietic stem cell transplantation.

Among the 309 patients, 256 (82.8%) experienced EBV reactivation within a year post-transplantation, and the median time to the first reactivation was 20 days (range, 3–244 days) after transplantation. The cumulative incidence in the first year is shown in Figure 1. The median initial EBV value at the time of reactivation was 7.18 × 10^3^ copies/ml (range, 1.01 × 10^3^–3.79 × 10^6^ copies/ml). The median time to achievement of peak EBV-DNA levels was 55 days (range, 10–548 days), with a median peak value of 1.53 × 10^5^ copies/ml (range, 2.14 × 10^3^–7.38 × 10^9^ copies/ml).

### 3.3 Analysis of risk factors for EBV reactivation

Univariate analysis identified risk factors for EBV reactivation, with variables exhibiting *p*-values < 0.05 advanced to multivariate analysis. As shown in Table 2, statistically significant variables in univariate analysis included donor type, stem cell source, ATG use in conditioning, HLA matching, and ATG dosage. In contrast, other conditioning agents—including busulfan, cyclophosphamide, fludarabine, acyclovir, thiotepa, and etoposide—showed no statistical significance. Given that ATG use and ATG dosage represent distinct data types for the same variable, both were included separately in the multivariate model. The final model with superior goodness-of-fit was selected using the Hosmer-Lemeshow test (Table 3), revealing ATG use (OR = 5.571, *p* < 0.001) as an independent risk factor.

**Table 2:** Univariate analysis of risk factors for post-transplant EBV reactivation.

Variables	EBV reactivated (*n* = 256)	EBV not reactivated (*n* = 53)	Univariate analysis	
			Z or *χ*^2^	*P*
Age, years, median (range)	2.9 (0.3–14.8)	3.1 (0.4–14.2)	−0.025	0.980
Dose of ATG, mg/kg, median (range)	7.44 (0.00–13.85)	6.82 (0.00–12.10)	−2.666	0.008
Sex, *n* (%)				
Men	177 (69.1)	41 (77.4)	1.427	0.232
Women	79 (30.9)	5 (22.6)		
Disease, *n* (%)				
Thalassemia	89 (34.8)	12 (22.6)	3.030	0.220
PID	110 (43.0)	28 (52.8)		
Other disease^a^	57 (22.3)	13 (24.5)		
Donor type, *n* (%)				
Unrelated	183 (71.5)	27 (50.9)	8.509	0.004
Related	73 (28.5)	26 (49.1)		
Stem cell source, *n* (%)				
PB	246 (96.1)	43 (81.1)	13.860	<0.001
Others^b^	10 (3.9)	10 (18.9)		
Donor-recipient ABO compatibility, *n* (%)				
Matched	99 (38.7)	27 (50.9)	2.738	0.098
Mismatched	157 (61.3)	26 (49.1)		
Use of ATG in conditioning, *n* (%)				
Yes	229 (89.5)	28 (52.8)	42.077	<0.001
No	27 (10.5)	25 (47.2)		
HLA matching, *n* (%)				
Matched	128 (50.0)	17 (32.1)	5.664	0.017
Mismatched	128 (50.0)	36 (67.9)		
EBV serostatus of recipient before transplantation, *n* (%)^c^				
IgG−	42 (16.9)	6 (12.2)	0.593	0.785
IgG±	12 (4.8)	2 (4.1)		
IgG+	195 (78.3)	41 (83.7)		
Pre-transplant EBV PCR, *n* (%)				
Positive	82 (32.0)	12 (22.6)	1.829	0.193
Negative	174 (68.0)	41 (77.4)		
Pre-transplant CMV PCR, *n* (%)				
Positive	22 (8.6)	6 (11.3)	0.134	0.714
Negative	234 (91.4)	47 (88.7)		
Acute graft-versus-host disease, *n* (%)				
Positive	166 (85.6)	90 (78.3)	2.712	0.100
Negative	28 (14.4)	25		

PID, primary immunodeficiency disease; PB, peripheral blood.

^a^ Other disease include leukemia, aplastic anemia, propionic acidemia, hemophagocytic lymphohistiocytosis, myelodysplastic syndrome, and myeloid sarcoma.

^b^ Others include the bone marrow, umbilical cord blood, peripheral blood + umbilical cord blood, bone marrow + umbilical cord blood, and peripheral blood + bone marrow.

^c^ No information on EBV serostatus of recipient before transplantation was available for 11 patients.

**Table 3:** Multivariate analysis of risk factors for post-transplant EBV reactivation.

Variables	Multivariate analysis		
	Exp (B)	95% CI	*p*
Donor type			0.999
Unrelated	1		
Related	1.001	0.456–2.197	
Stem cell source			0.231
PB	1		
Others	0.507	0.167–1.541	
Use of ATG in conditioning			<0.001
No	1		
Yes	5.571	2.261–13.723	
HLA matching			0.347
Matched	1		
Mismatched	1.411	0.689–2.890	

### 3.4 Incidence of PTLD after EBV reactivation

**Figure 2:**
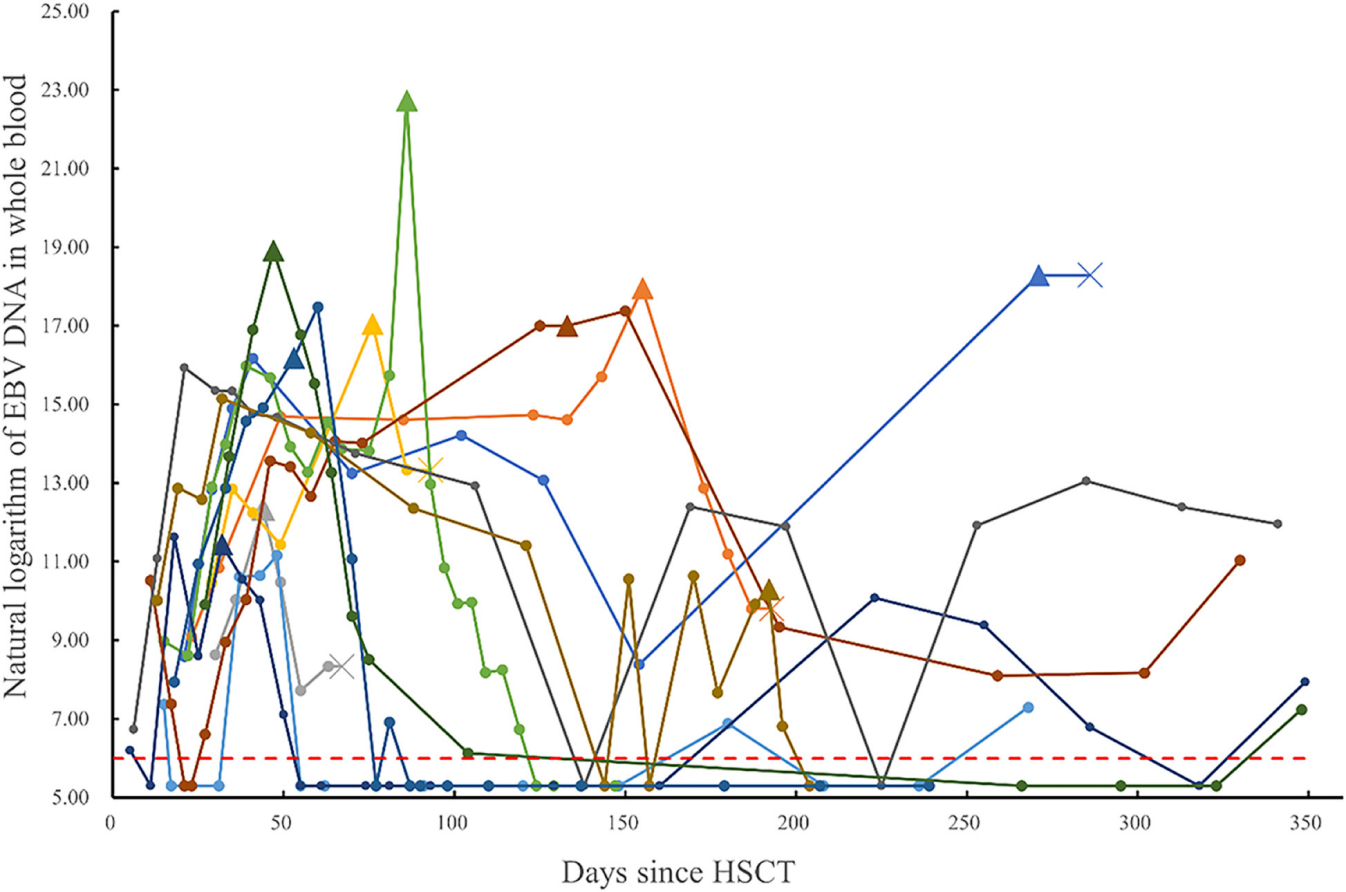
Monitoring of post-transplant whole blood EBV-DNA (natural logarithm) in patients diagnosed with PTLD (data points below the detection threshold are recorded as natural logarithm of 200 copies/ml. Different colored lines represent different patients, indicates the status of each monitoring point, ×indicates the death of the patient, ▴ represents the nearest monitoring point before Rituximab.

Among the 256 post-transplant patients who exhibited EBV reactivation, 11 were diagnosed with probable PTLD, and 1 with proven PTLD. A summary of their clinical data is provided in the Supplementary Material. The median time to PTLD diagnosis post-transplantation was 66 days (range, 23–272 days). The whole-blood EBV PCR monitoring results for these 12 patients are depicted in Figure 2. The median time to EBV reactivation in the PTLD group was 20 days (range, 11–37 days), which was not significantly different from the non-PTLD group, with a median time of 20 days (range, 3–244 days; *P* = 0.444). Similarly, the median time to reach peak EBV load was not statistically different between the PTLD group (54 days; range, 18–271 days) and the non-PTLD group (55 days; range, 10–548 days; *P* = 0.986). However, there was a significant difference in the peak EBV levels detected by PCR between the PTLD and non-PTLD groups. The PTLD group exhibited peak EBV levels of 3.01 × 10^7^ copies/ml (range, 6.99 × 10^4^–7.38 × 10^9^ copies/ml), whereas the non-PTLD group showed peak levels of 1.45 × 10^5^ copies/ml (range, 2.14 × 10^3^–4.66 × 10^8^ copies/ml; *P* < 0.001). Additionally, there was a statistically significant difference in the blood lactic dehydrogenase levels at the time of peak EBV PCR between the non-PTLD and PTLD groups (Z = −2.702, *P* = 0.007).

### 3.5 Analysis of risk factors for PTLD occurrence after EBV reactivation

Outcomes of univariate and multivariate analyses for PTLD incidence are presented in Tables 4, 5, respectively. Variables not listed in Table 4—including alternative conditioning regimens, hematopoietic recovery status, GVHD prophylaxis, lymphocyte subpopulation counts, absolute lymphocyte counts, and serial measurements of immunoglobulins (IgM, IgA, IgG), complements (C3, C4), and blood LDH at reactivation—showed no significant association with PTLD in univariate analysis. Subsequent univariate analysis identified grade III–IV aGVHD, day-30 blood C3, day-30 IgE, day-30 IgM, and day-100 absolute/relative lymphocyte counts as significant risk factors (*p* < 0.05). Due to the strong correlation between CD19 + lymphocyte counts and rituximab use, we excluded patients receiving rituximab within 100 days post-transplant. This revealed no significant differences in day-100 CD19 + counts between groups. We therefore derived highest absolute/relative CD19 + counts within 100 days as new variables, but these likewise showed no intergroup differences. Multivariate analysis (Table 5) confirmed only grade III–IV aGVHD as an independent risk factor for PTLD following EBV reactivation (OR = 13.730, *p* = 0.028). In our study, 21 patients received rituximab, among whom 11 were diagnosed with PTLD. Six patients received 1–4 doses of rituximab at a median of 52 days (range, 31–171 days) due to significant EBVemia, while 3 patients were treated for autoimmune hemolytic anemia and 1 for immune thrombocytopenia.

**Table 4:** Univariate analysis of risk factors for progression to post-transplant lymphoproliferative disorder (PTLD) after EBV reactivation.

Variables	EBV reactivation without PTLD (*n* = 244)	EBV reactivation with PTLD (*n* = 12)	Univariate analysis	
			Z or *χ*^2^	*p*
Sex, *n* (%)				
Male	168 (68.9)	9 (75.0)	0.017	0.897
Female	76 (31.1)	3 (25.0)		
Disease, *n* (%)				
Thalassemia	86 (35.2)	3 (25.0)	2.596	0.278
PID	102 (41.8)	8 (66.7)		
Other disease	56 (23.0)	1 (8.3)		
Donor type, *n* (%)				
Unrelated	174 (71.3)	9 (75.0)	0.000	1.000
Related	70 (28.7)	3 (25.0)		
Stem cell source, *n* (%)				
PB	236 (96.7)	10 (83.3)	2.477	0.116
Others	8 (3.3)	2 (16.7)		
Donor-recipient ABO compatibility, *n* (%)				
Matched	95 (38.9)	4 (33.3)	0.007	0.932
Mismatched	149 (61.1)	8 (66.7)		
Use of ATG in conditioning, *n* (%)				
No	27 (11.1)	0 (0.0)	0.543	0.461
Yes	217 (88.9)	12 (100.0)		
GVHD preventing strategy, *n* (%)				
CsA	12 (4.9)	0 (0.0)	2.939	0.350
CsA + MTX	40 (16.4)	0 (0.0)		
CsA + MMF	122 (50.0)	9 (75.0)		
CsA + MTX + MMF	70 (28.7)	3 (25.0)		
HLA matching, *n* (%)				
Matched	125 (51.2)	3 (25.0)	3.148	0.076
Mismatched	119 (48.8)	9 (75.0)		
EBV serostatus of recipient before transplantation, *n* (%)^a^				
IgG−	40 (16.9)	2 (16.7)	0.118	1.000
IgG±	12 (4.3)	0 (0.0)		
IgG+	185 (78.1)	10 (83.3)		
Pre-transplant EBV PCR, *n* (%)				
Positive	79 (32.4)	3 (25.0)	0.047	0.828
Negative	165 (67.6)	9 (75.0)		
Pre-transplant CMV PCR, *n* (%)				
Positive	21 (8.6)	1 (8.3)	0.000	1.000
Negative	223 (91.4)	11 (91.7)		
Acute graft-versus-host disease, *n* (%)				
Grade 0	90^a^ (36.9)	1^a^ (8.3)	9.866	0.005
Grade I–II	138^a^ (56.6)	7^a^ (58.3)		
Grade III–IV	16^b^ (6.6)	4^b^ (33.3)		
Age, years, median (range)	2.8 (0.3–14.8)	2.8 (0.8–7.9)	−0.170	0.865
Dose of ATG, mg/kg, median (range)	7.41 (0.00–13.85)	7.55 (5.45–11.38)	−0.785	0.432
Complement C3 in blood on day 30, g/L, median (range)^b^	0.86 (0.15–1.79)	0.71 (0.48–1.31)	−2.433	0.015
IgE in blood on day 30, IU/ml, median (range)^b^	15.1 (0.0–1,253.0)	43.6 (5.1–902.0)	−2.003	0.045
IgM in blood on day 30, g/L, median (range)^b^	0.59 (0.11–5.13)	0.31 (0.10–1.12)	−2.446	0.014
Relative count of CD19 + lymphocyte on day100, %, median (range)	1.26 (0.04–26.92)	0.27 (0.00–25.44)	−2.250	0.024
Absolute count of CD19 + lymphocyte on day100, cells/ul, median (range)	18.16 (0.30–582.84)	2.37 (0.00–870.12)	−0.832	0.021
Highest relative count of CD19 + lymphocyte within 100 days since HSCT, %, median (range)	2.77 (0.00–61.00)	5.17 (0.00–25.44)	−0.501	0.616
Highest absolute count of CD19 + lymphocyte within 100 days since HSCT, cells/ul, median (range)	29.97 (0.97–982.03)	23.67 (1.13–870.12)	−0.487	0.626

Day XX represents the day after allo-HSCT; LDH, lactic dehydrogenase.

^a^ No information was available on EBV serostatus of recipient before transplantation for 7 patients.

^b^ No information on IgM, IgE, and Complement C3 levels was available for 7 patients.

CsA, Cyclosporin; MTX, methotrexate; MMF, mycophenolate mofetil.

**Table 5:** Multivariate analysis of risk factors for progression to PTLD after EBV reactivation.

Variables	Multivariate analysis		
	Exp (B)	95% CI	*p*
Acute graft vs. host disease, *n* (%)			
Grade 0	1		0.078
Grade I–II	4.611	0.544–39.065	0.161
Grade III–IV	13.730	1.324–142.363	0.028
Complement C3 in blood on day 30, g/L, median (range)	0.179	0.006–5.150	0.699
IgE in blood on day 30, IU/ml, median (range)	1.001	0.999–1.004	0.340
IgM in blood on day 30, g/L, median (range)	0.104	0.008–1.376	0.086

### 3.6 Diagnostic value of whole-blood EBV PCR for PTLD

**Figure 3:**
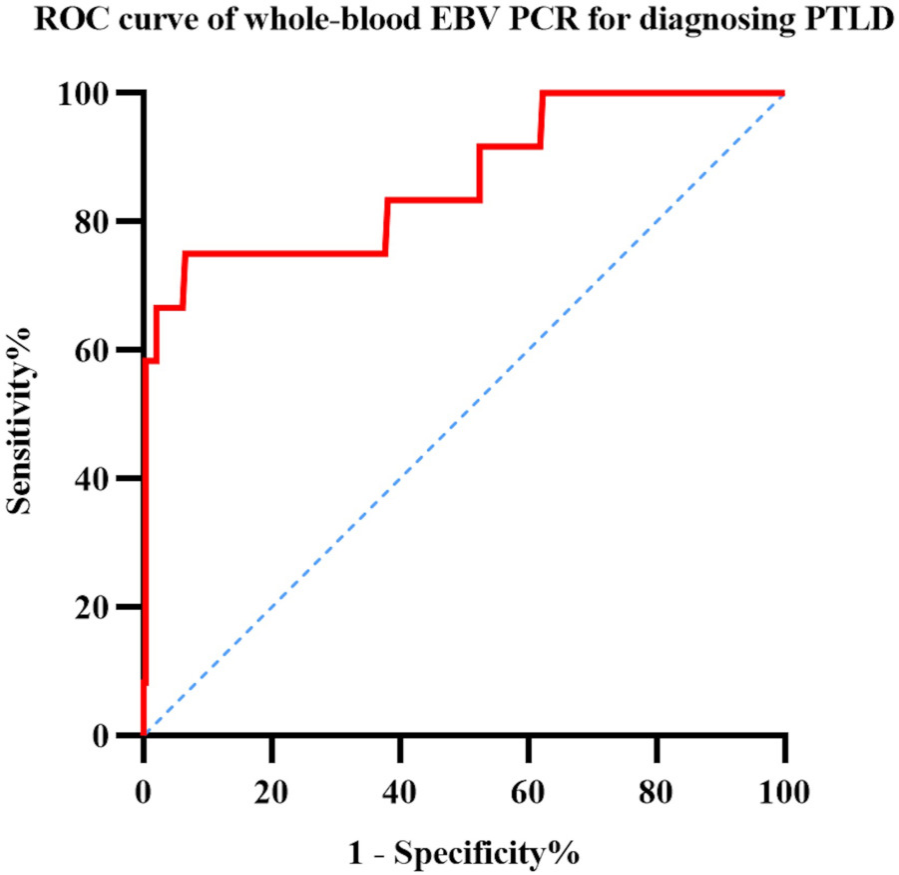
ROC curve analysis of sensitivity and specificity for diagnosing PTLD based on whole-blood EBV PCR.

The ROC curves were analyzed to determine the optimal threshold for diagnosing PTLD based on the maximum EBV viral DNA load; the results are shown in Figure 3. This analysis demonstrated that when the whole-blood EBV load was 3.755 × 10^6^ copies/ml, the area under the ROC curve was largest (0.864), with a sensitivity of 0.75 and specificity of 0.934.

### 3.7 The effect of ATG on *T* cell immune reconstitution after transplantation

In accordance with the application of ATG in conditioning regimens, participants were categorized into two distinct cohorts: the non-ATG usage group and the ATG group. A comparative analysis of *T* cell counts between these two groups was conducted on days 15, 30, 100, and 180, with the findings presented in Table 6. Notably, significant differences in the absolute counts of CD3+, CD8+, and CD4+ *T* cells were observed between the ATG group and the non-usage group on day 15. Subsequently, from day 30 to day 180, no significant differences were detected in the absolute counts of CD3+ *T* cells and CD8+ *T* cells between the two cohorts. In contrast, a statistically significant disparity in CD4+ *T* cell counts persisted between the groups throughout this period.

**Table 6:** The results of *T* cell reconstruction at different time after transplantation using ATG or not.

Days after transplantation	Classification of *T* cells	Non-ATG group (cells/μl)	ATG group (cells/μl)	Z	*P*
Day15 (*n* = 295)	CD3+	1,172.9 (33.5–7,210.3)	358.24 (0.5–5,293.1)	−3.583	<0.001
	CD4+	342.1 (19.1–2,592.77)	87.5 (0.5–1,588.7)	−5.975	<0.001
	CD8+	448.1 (4.5–6,524.5)	200.9 (0.5–4,174.6)	−2.133	0.033
Day30 (*n* = 299)	CD3+	939.4 (206.8–4,831.2)	737.1 (2.2–5,037.3)	−1.485	0.138
	CD4+	305.5 (67.8–746.5)	105.8 (1.47–3,540.8)	−6.502	<0.001
	CD8+	557.7 (76.85–4,318.8)	492.6 (1.17–4,823.0)	−0.018	0.986
Day100 (*n* = 269)	CD3+	1,360.2 (280.6–3,077.3)	1,230.7 (180.3–7,414.7)	−0.071	0.944
	CD4+	398.0 (103.0–902.1)	178.3 (15.7–1,101.0)	−5.021	<0.001
	CD8+	833.0 (42.8–2,241.1)	885.0 (114.7–5,734.9)	−1.899	0.058
Day180 (*n* = 218)	CD3+	1,609.6 (10.3–3,832.18)	1,407.5 (130.5–6,613.5)	−0.183	0.855
	CD4+	513.2 (102.6–988.6)	199.6 (35.2–3,187.7)	−6.135	<0.001
	CD8+	1,007.6 (33.1–2,454.3)	1,060.2 (54.8–5,436.3)	−1.879	0.060

### 3.8 Analysis of dynamic parameters of whole-blood EBV load for predicting PTLD occurrence

The dynamic parameters of viral DNA, such as doubling time, have been shown to assist in the management of CMV infections in allogeneic HSCT (20). According to a study by Solano et al. (6), dynamic parameter analysis of the plasma EBV viral load might not predict the occurrence of PTLD. They speculated that whole-blood EBV viral load may have some significance. We assessed our center's whole-blood EBV monitoring results using the following formula: doubling time (dt) = (t2—t1) × log (2)/log (q2/q1), where q1 and t1 are the EBV DNA loads (copies/ml) at the time of the first positive PCR (in days), and q2 and t2 are the EBV DNA loads at the time of the second positive PCR. The time interval between t1 and t2 was less than 10 days, and an increase in the EBV DNA load by at least three or more times was considered relevant and used to evaluate dt. Among 289 EBVemia patients, excluding 13 children for whom calculations were not possible, a statistical difference was observed in the occurrence of EBV doubling between the PTLD and non-PTLD groups (*χ*^2^ = 5.735; *P* = 0.017), but no statistical differences were observed in the doubling time (dt) between the two groups (Z = −0.74; *P* = 0.459).

### 3.9 Follow-up of pediatric patients who underwent transplantation

**Figure 4:**
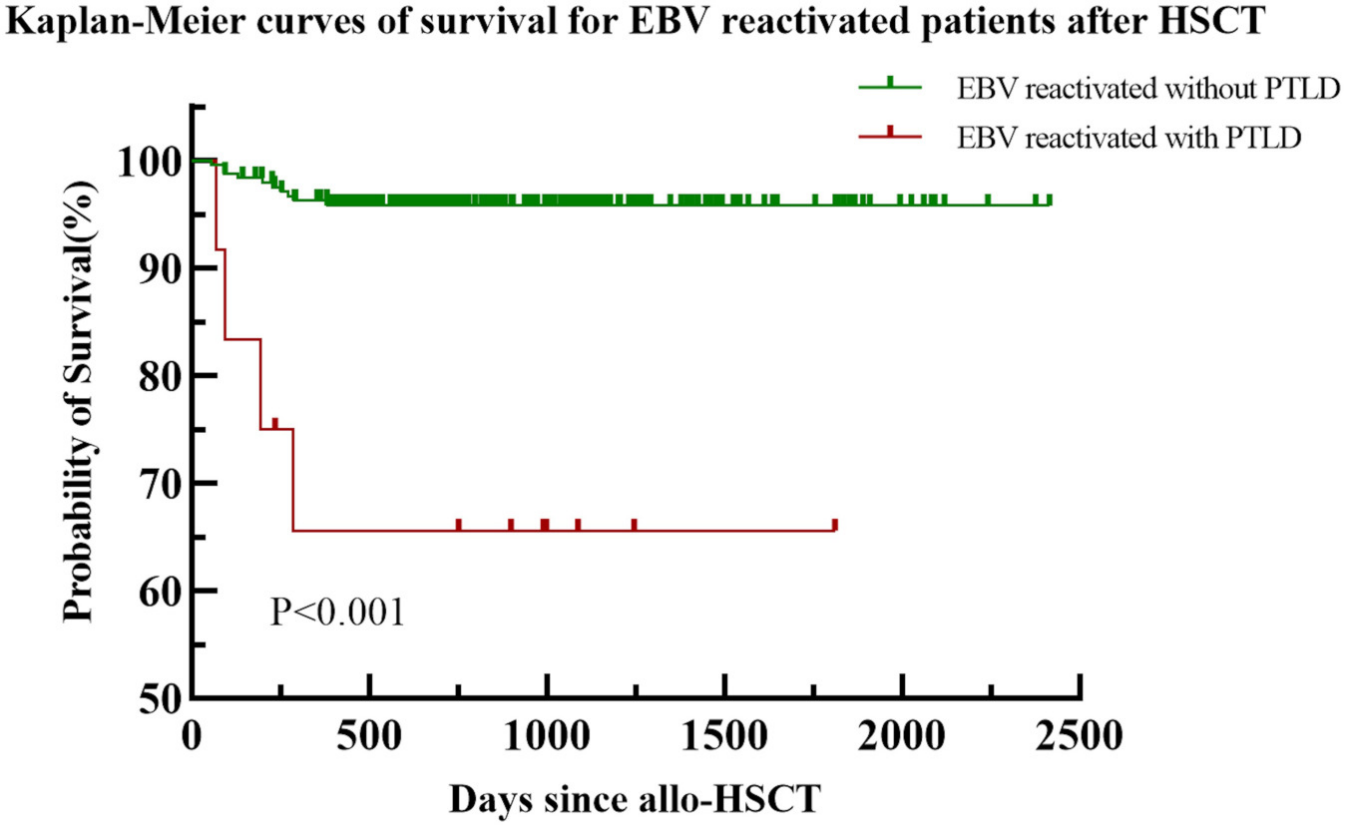
Survival curves of 256 patients underwent allo-HSCT with EBV reactivation.

The median follow-up period of the 309 pediatric patients was 750 days (range, 53–2414 days). Sixteen pediatric patients died, with a median time to death of 196 days (range, 53–850 days). Common causes of death after transplantation included infection-related complications (11 cases; 68.75%), severe gastrointestinal GVHD (2 cases; 12.5%), intracranial hemorrhage (1 case; 6.25%), bronchiolitis obliterans (1 case; 6.25%), and immune cytopenia (1 case; 6.25%). The follow-up status of the 256 patients with EBV reactivation is shown in Figure 4, There is a significant statistical difference in survival with PTLD and without PTLD (*P* < 0.001). Among the 12 patients with PTLD, eight children survived, the clinical information of the four deceased children with PTLD is shown in Supplementary Material.

## 4 Discussion

In this study, we have reported the incidence of EBV reactivation and PTLD in pediatric recipients of allogeneic HSCT. We analyzed the risk factors for EBV reactivation in 309 pediatric patients and those for progression to PTLD in 256 patients with EBV reactivation after transplantation. We found that ATG was the independent risk factor for EBV reactivation after transplantation. The independent risk factor for PTLD after EBV reactivation was grade III–IV aGVHD. Using the whole-blood EBV DNA load for PTLD diagnosis, 3.755 × 10^6^ copies/ml was determined to be the optimal threshold having the highest area under the ROC curve, indicating high specificity, which served as a suggestive threshold for initiating rituximab treatment at our center.

Our center observed a moderately elevated EBV reactivation rate compared to other transplant centers. This discrepancy may stem from inter-institutional variations in diagnostic criteria, specimen types (whole blood vs. serum/plasma), and detection thresholds. Whole blood EBV PCR demonstrates higher clinical sensitivity than plasma/serum testing—a critical methodological consideration (21, 22). Additionally, as delineated in established guidelines (2) and corroborated by other studies (14, 23, 24), as well as our own research findings, the use of anti-thymocyte globulin (ATG) emerges as an independent risk factor for EBV reactivation following transplantation. ATG administration results in the depletion of CD8+ T cells, which are pivotal in the suppression of viral reactivation. Our data reveal that the absolute count of CD8+ T cells on day 15 is markedly higher in patients who did not receive ATG as part of their conditioning regimen compared to those who did (Z = −2.133, *P* = 0.033). In conclusion, given that the majority of stem cell grafts at our center are derived from unrelated donors, the utilization rate of ATG is notably high at 83.2%. This contributes to the elevated incidence and rapid onset of EBV reactivation observed in our patient population.

The guidelines on PTLD (2) propose that *T* cell depletion *in vivo* or *in vitro* is a risk factor for PTLD. However, our research shows that the application of ATG has no significant impact on the progression of PTLD after EBV reactivation. Firstly, this may be due to the collinearity between EBV reactivation and PTLD after hematopoietic stem cell transplantation, which may affect the analysis of risk factors. Additionally, this may be due to the rapid reconstruction of CD8+ *T* cells after HSCT (25), which precedes EBV carcinogenic proliferation; The median occurrence time of PTLD is generally 2–4 months after transplantation (2). In our center, the median occurrence time of PTLD is 66 days after transplantation. Although our data shows a statistically significant difference in CD8+ *T* cell absolute counts between the ATG group and the non ATG group on day 15, there is no statistically significant difference in CD8+ *T* cell absolute count on day 30 between the two groups (Z = −0.018, *P* = 0.986), indicating that the use of ATG before the occurrence of PTLD has no significant effect on CD8+ *T* cell reconstruction. In addition, the use of ATG can reduce the incidence of acute and chronic GVHD (26), and the reduction of immunosuppressive therapy after transplantation is beneficial for immune monitoring and immune defense of the body after transplantation.

In our study, we observed a high incidence of EBV reactivation coupled with a surprisingly low rate of PTLD. Consequently, we conducted a thorough analysis of the factors associated with PTLD progression following EBV reactivation and synthesized these findings with the outcomes of post-transplant immune reconstitution. The development of grade III–IV aGVHD requires intensive immunosuppression, which may precipitate a proinflammatory cytokine storm capable of impairing targeted immune reconstitution and thus creating a conducive environment for PTLD development. Striking a balance between viral reactivation and the intensity of GVHD poses a significant therapeutic challenge. We noted that PTLD patients exhibit elevated LDH levels at the zenith of EBV-DNA load, a phenomenon attributed to the characteristic uncontrolled proliferation of highly motile malignant cells. While elevated LDH levels are a common feature in PTLD following solid organ transplantation (27); however, further research is required to determine the diagnostic value of elevated LDH levels for PTLD after HSCT.

A study by the Institute of Hematology at Peking University proposed that a low absolute count of CD8+ *T* cells on the 30th day is a risk factor for PTLD (28). However, in our study, the absolute and relative counts of lymphocytes on the 15th and 30th days did not show statistical significance in progressing to PTLD after EBV reactivation. This may be due to the fact that our transplant population is children, and due to differences in thymic function compared to adults, the influence of age on *T* cell reconstruction after hematopoietic stem cell transplantation cannot be ignored. Another study by the Institute of Hematology at Peking University (29) showed that the *T* cell subset of CD4-CD8- may interact with EBV reactivation. At 30, 90, and 180 days after haploid transplantation, the absolute count of CD4-CD8-*T* cells in EBV recipients was significantly lower than that in non EBV recipients. Burns’ study (30) showed that in patients with high EBV load, the proportion and quantity of CD27+ memory B cells significantly increased, expressing EBV latent transcripts with plasma cell phenotype associated with B cell growth and transformation, and frequently expressing proliferation marker Ki-67. EBV reactivation may drive the expansion of CD27+ B lymphoblasts with latent infection in peripheral blood. From the above research, it can be seen that the relationship between EBV reactivation, PTLD, and immune reconstitution is complex and not yet fully understood. Our study may require further research on the effects of immune reconstitution on EBV reactivation and progression to PTLD. Perhaps more accurate lymphocyte classification tests can yield richer results.

Due to the serious impact of PTLD on the survival prognosis of transplanted children, early identification and diagnosis are very important. Compared with pathological biopsy, monitoring the EBV-DNA load after transplantation is relatively easy and less traumatic. However, currently there is no unified test standard, and the recommended treatment thresholds vary from center to center. However, the combination of rituximab treatment and immunosuppressive therapy is currently the first-line treatment for PTLD, and the prognosis of about 70% of PTLD patients can be improved (2). Based on the results of ROC curve analysis, we recommend rituximab intervention if the whole-blood EBV load reaches 3.755 × 10^6^ copies/ml; similar to that observed in Solano's study (6) on the plasma EBV load, our findings showed that the doubling time of the whole-blood EBV load was not a significant predictive factor for PTLD.

Our study was limited by its single-center retrospective design. Further multicenter prospective studies are needed to validate the generalizability and reproducibility of the results. Multiple studies identify donor-recipient EBV serological mismatch as a significant risk factor for PTLD, with the EBV-seronegative recipient/EBV-seropositive donor combination conferring the highest risk (2, 11, 31), Mechanistically, donor EBV IgG positivity reflects latent infection, while recipient EBV IgG negativity indicates absent protective antibodies. Under post-transplant immunosuppression, this seromismatch potentiates uncontrolled EBV proliferation. However, our dataset lacked donor EBV serostatus because routine EBV serological testing was not included in donor screening protocols by the China Marrow Donor Program during the study period.

In conclusion, our study is limited by its retrospective a single-center design, but it helps to identify the patients have the risk for progression to PTLD after EBV reactivation since the incidence rate of PTLD is not high. While no consensus has been reached regarding the optimal sample type or EBV PCR threshold for initiating rituximab treatment. Our study gives the threshold of whole-blood EBV-DNA for rituximab treatment. Considering the high mortality of PTLD, it could be of great interest to study the risk factors for PTLD and threshold for rituximab treatment in a larger and multi-center cohort with unified standards of EBV PCR. Finally, regular monitoring of EBV PCR plays an important role after hematopoietic stem cell transplantation.

## Data Availability

The original contributions presented in the study are included in the article/Supplementary Material, further inquiries can be directed to the corresponding author.

## References

[1] Chang CM Yu KJ Mbulaiteye SM Hildesheim A Bhatia K. The extent of genetic diversity of Epstein–Barr virus and its geographic and disease patterns: a need for reappraisal. Virus Res. (2009) 143(2):209–21. 10.1016/j.virusres.2009.07.005 19596032 PMC2731007

[2] Styczynski J van der Velden W Fox CP Engelhard D de la Camara R Cordonnier C Management of Epstein–Barr virus infections and post-transplant lymphoproliferative disorders in patients after allogeneic hematopoietic stem cell transplantation: sixth European conference on infections in leukemia (ECIL-6) guidelines. Haematologica. (2016) 101(7):803–11. 10.3324/haematol.2016.144428 27365460 PMC5004459

[3] Fujimoto A Hiramoto N Yamasaki S Inamoto Y Uchida N Maeda T Risk factors and predictive scoring system for post-transplant lymphoproliferative disorder after hematopoietic stem cell transplantation. Biol Blood Marrow Transplant. (2019) 25(7):1441–9. 10.1016/j.bbmt.2019.02.016 30794929

[4] Kołodziejczak M Gil L de la Camara R Styczyński J. Impact of donor and recipient Epstein–Barr virus serostatus on outcomes of allogeneic hematopoietic cell transplantation: a systematic review and meta-analysis. Ann Hematol. (2021) 100(3):763–77. 10.1007/s00277-021-04428-9 33491135 PMC7914248

[5] Kang HM Kim SK Lee JW Chung NG Cho B. Efficacy of low dose antithymocyte globulin on overall survival, relapse rate, and infectious complications following allogeneic peripheral blood stem cell transplantation for leukemia in children. Bone Marrow Transplant. (2021) 56(4):890–9. 10.1038/s41409-020-01121-9 33199818

[6] Solano C Mateo EM Pérez A Talaya A Terol MJ Albert E Epstein–Barr virus DNA load kinetics analysis in allogeneic hematopoietic stem cell transplant recipients: is it of any clinical usefulness? J Clin Virol. (2017) 97:26–32. 10.1016/j.jcv.2017.10.016 29096390

[7] Wareham NE Mocroft A Sengeløv H Da Cunha-Bang C Gustafsson F Heilmann C The value of EBV DNA in early detection of post-transplant lymphoproliferative disorders among solid organ and hematopoietic stem cell transplant recipients. J Cancer Res Clin Oncol. (2018) 144(8):1569–80. 10.1007/s00432-018-2674-9 29804164 PMC11813311

[8] Wang H Zhang TT Qi JQ Chu TT Miao M Qiu HY Incidence, risk factors, and clinical significance of Epstein–Barr virus reactivation in myelodysplastic syndrome after allogeneic haematopoietic stem cell transplantation. Ann Hematol. (2019) 98(4):987–96. 10.1007/s00277-019-03603-3 30715567

[9] Ru Y Zhang X Song T Ding Y Zhu Z Fan Y Epstein–Barr virus reactivation after allogeneic hematopoietic stem cell transplantation: multifactorial impact on transplant outcomes. Bone Marrow Transplant. (2020) 55(9):1754–62. 10.1038/s41409-020-0831-7 32066862

[10] Kalra A Roessner C Jupp J Williamson T Tellier R Chaudhry A Epstein-Barr virus DNAemia monitoring for the management of post-transplant lymphoproliferative disorder. Cytotherapy. (2018) 20(5):706–14. 10.1016/j.jcyt.2018.02.367 29580864

[11] Uhlin M Wikell H Sundin M Blennow O Maeurer M Ringden O Risk factors for Epstein–Barr virus-related post-transplant lymphoproliferative disease after allogeneic hematopoietic stem cell transplantation. Haematologica. (2014) 99(2):346–52. 10.3324/haematol.2013.087338 24056821 PMC3912966

[12] Stocker N Labopin M Boussen I Paccoud O Bonnin A Malard F Pre-emptive rituximab treatment for Epstein–Barr virus reactivation after allogeneic hematopoietic stem cell transplantation is a worthwhile strategy in high-risk recipients: a comparative study for immune recovery and clinical outcomes. Bone Marrow Transplant. (2020) 55(3):586–94. 10.1038/s41409-019-0699-6 31562397

[13] Kinch A Hallböök H Arvidson J Sällström K Bondeson K Pauksens K. Long-term outcome of Epstein–Barr virus DNAemia and PTLD with the use of preemptive rituximab following allogeneic HSCT. Leuk Lymphoma. (2018) 59(5):1172–9. 10.1080/10428194.2017.1365860 28831836

[14] Gao XN Lin J Wang LJ Li F Li HH Wang SH Risk factors and clinical outcomes of Epstein–Barr virus DNAemia and post-transplant lymphoproliferative disorders after haploidentical and matched-sibling PBSCT in patients with hematologic malignancies. Ann Hematol. (2019) 98(9):2163–77. 10.1007/s00277-019-03742-7 31243569

[15] Salas MQ Prem S Remberger M Lam W Kim DDH Michelis FV High incidence but low mortality of EBV-reactivation and PTLD after alloHCT using ATG and PTCy for GVHD prophylaxis. Leuk Lymphoma. (2020) 61(13):3198–208. 10.1080/10428194.2020.1797010 32715815

[16] Lindsay J Othman J Yong MK Ritchie D Chee L Tay K Dynamics of Epstein–Barr virus on post-transplant lymphoproliferative disorders after antithymocyte globulin-conditioned allogeneic hematopoietic cell transplant. Transplant Infect Dis. (2021) 23(5):e13719. 10.1111/tid.13719 34453768

[17] Kania SP Silva JMF Charles OJ Booth J Cheung SYA Yates JWT Epstein–Barr virus reactivation after paediatric haematopoietic stem cell transplantation: risk factors and sensitivity analysis of mathematical model. Front Immunol. (2022) 13:903063. 10.3389/fimmu.2022.903063 35903096 PMC9314642

[18] Landgren O Gilbert ES Rizzo JD Socié G Banks PM Sobocinski KA Risk factors for lymphoproliferative disorders after allogeneic hematopoietic cell transplantation. Blood. (2009) 113(20):4992–5001. 10.1182/blood-2008-09-178046 19264919 PMC2686146

[19] Liu J Bian Z Wang X Xu LP Fu Q Wang C Inverse correlation of V*δ*2(+) T-cell recovery with EBV reactivation after haematopoietic stem cell transplantation. Br J Haematol. (2018) 180(2):276–85. 10.1111/bjh.15037 29270985

[20] Giménez E Muñoz-Cobo B Solano C Amat P Navarro D. Early kinetics of plasma cytomegalovirus DNA load in allogeneic stem cell transplant recipients in the era of highly sensitive real-time PCR assays: does it have any clinical value? J Clin Microbiol. (2014) 52(2):654–6. 10.1128/JCM.02571-13 24478505 PMC3911364

[21] Rzepka M Depka D Gospodarek-Komkowska E Bogiel T. Diagnostic value of whole-blood and plasma samples in Epstein–Barr virus infections. Diagnostics (Basel). (2023) 13(3):476. 10.3390/diagnostics13030476 36766581 PMC9914079

[22] Gao W Wu J Han Y Fu X. Study on diagnostic value of results of EB virus nucleic acid quantification in whole blood lymphocyte and serum in children with EB virus infection. Cytokine. (2022) 155:155902. 10.1016/j.cyto.2022.155902 35561585

[23] Massoud R Gagelmann N Fritzsche-Friedland U Zeck G Heidenreich S Wolschke C Comparison of immune reconstitution between anti-T-lymphocyte globulin and posttransplant cyclophosphamide as acute graft-versus-host disease prophylaxis in allogeneic myeloablative peripheral blood stem cell transplantation. Haematologica. (2022) 107(4):857–67. 10.3324/haematol.2020.271445 33832208 PMC8968885

[24] Walker I Panzarella T Couban S Couture F Devins G Elemary M Pretreatment with anti-thymocyte globulin versus no anti-thymocyte globulin in patients with haematological malignancies undergoing haemopoietic cell transplantation from unrelated donors: a randomised, controlled, open-label, phase 3, multicentre trial. Lancet Oncol. (2016) 17(2):164–73. 10.1016/S1470-2045(15)00462-3 26723083

[25] Minculescu L Marquart HV Ryder LP Andersen NS Schjoedt I Friis LS Improved overall survival, relapse-free-survival, and less graft-vs.-host-disease in patients with high immune reconstitution of TCR gamma Delta cells 2 months after allogeneic stem cell transplantation. Front Immunol. (2019) 10:1997. 10.3389/fimmu.2019.01997 31507601 PMC6714591

[26] Chang YJ Wu DP Lai YR Liu QF Sun YQ Hu J Antithymocyte globulin for matched sibling donor transplantation in patients with hematologic malignancies: a multicenter, open-label, randomized controlled study. J Clin Oncol. (2020) 38(29):3367–76. 10.1200/JCO.20.00150 32650683

[27] Knight JS Tsodikov A Cibrik DM Ross CW Kaminski MS Blayney DW. Lymphoma after solid organ transplantation: risk, response to therapy, and survival at a transplantation center. J Clin Oncol. (2009) 27(20):3354–62. 10.1200/JCO.2008.20.0857 19451438

[28] Xu LP Zhang CL Mo XD Zhang XH Chen H Han W Epstein–Barr virus-related post-transplantation lymphoproliferative disorder after unmanipulated human leukocyte antigen haploidentical hematopoietic stem cell transplantation: incidence, risk factors, treatment, and clinical outcomes. Biol Blood Marrow Transplant. (2015) 21(12):2185–91. 10.1016/j.bbmt.2015.07.035 26253005

[29] Bian Z Liu J Xu LP Chang YJ Wang Y Zhang XH Association of Epstein–Barr virus reactivation with the recovery of CD4/CD8 double-negative T lymphocytes after haploidentical hematopoietic stem cell transplantation. Bone Marrow Transplant. (2017) 52(2):264–9. 10.1038/bmt.2016.238 27797369

[30] Burns DM Tierney R Shannon-Lowe C Croudace J Inman C Abbotts B Memory B-cell reconstitution following allogeneic hematopoietic stem cell transplantation is an EBV-associated transformation event. Blood. (2015) 126(25):2665–75. 10.1182/blood-2015-08-665000 26450987 PMC4732759

[31] Kalra A Roessner C Jupp J Williamson T Tellier R Chaudhry A Risk factors for post-transplant lymphoproliferative disorder after thymoglobulin-conditioned hematopoietic cell transplantation. Clin Transplant. (2018) 32(1):e13150. 10.1111/ctr.13150 29114932

