# Supplementary material for: Analysis of risk factors for Epstein–Barr virus reactivation and progression to post-transplant lymphoproliferative disorder in pediatric patients undergoing allogeneic hematopoietic stem cell transplantation
Source: Front Pediatr. 2025 Jul 17;13:1627990. doi: 10.3389/fped.2025.1627990 (PMC12310745; doi:10.3389/fped.2025.1627990)
Supplement: Supplementary file 1 [file Table1.docx]

Table 1 clinical data of 12 PTLD patients

| Patient | Primary disease | Time of diagnosing PTLD after transplantation | Clinical presentation and imaging findings | Fever lasting time (days) | Location of lymph node enlargement | Blood routine examination | immunosuppressive therapy before PTLD | Time of immunosuppressive therapy reduction | Other fluid specimens PCR of EBV | Ferritin (ng/ml) | Relative count of CD19+ lymphocytes (%) | Time of Rituximab treatment |
| --- | --- | --- | --- | --- | --- | --- | --- | --- | --- | --- | --- | --- |
| 1 | WAS | D272 | Fever, lymphadenopathy, hepatosplenomegaly, diarrhea, gastrointestinal bleeding, intestinal obstruction | 23 | pretracheal, abdominal and retroperitoneal lymph nodes | Anemia, thrombocytopenia | Tacrolimus, prednisone | D280 | Cerebrospinal fluid 2.31*10^4^ | >7500 | 52.01 | D281 |
| 2 | SCID | D162 | Fever, convulsion, lymphadenopathy, gastrointestinal bleeding, intestinal obstruction | 28 | Mesenteric lymph nodes | Anemia, leukopenia, thrombocytopenia | Tacrolimus, methylprednisolone | D159 | Cerebrospinal fluid 1.75*10^6^ | 5099 | 10.74 | D172, D179, D186 |
| 3 | XLP | D45 | Fever, pericardial effusion, hepatosplenomegaly, ascites, pleural effusion | 22 | Cervical and axillary lymph nodes | Anemia, thrombocytopenia | Tacrolimus | D46 |  | 1674 |  | D45, D52, D59 |
| 4 (proven) | FA | D78 | Fever, convulsion, elevated ferritin, lymphadenopathy | 29 | Mesenteric and Right axillary lymph nodes | Anemia, leukopenia, thrombocytopenia | Tacrolimus, prednisone | D75 D82 | Cerebrospinal fluid 6.19*10^5^ | 5127 | 46.47 | D80, D87 |
| 5 | WAS | D48 | Hepatosplenomegaly, lymphadenopathy, abdominal distension. | 0 | Mesenteric, Bilateral axillary, inguinal and left cervical lymph nodes | Anemia, leukopenia, thrombocytopenia | Tacrolimus, mycophenolate mofetil, prednisone | D48 | Sputum 7.07*10^5^ | 1485 | 1.36 | D49, D58, D65，D72 |
| 6 | WAS | D88 | Fever, abdominal pain, altered consciousness, lymphadenopathy, Thoracic effusion, pericardial effusion, abdominal effusion | 19 | Superficial lymph nodes | Anemia, leukopenia, thrombocytopenia | Tacrolimus, mycophenolate mofetil | D86 | Cerebrospinal fluid 9.87*10^2^  Sputum 1.45*10^5^  Hydrothorax 1.19*10^4^ | 2252 | 12.81 | D88, D95, D102, D109 |
| 7 | HIGM | D34 | Hepatomegaly, abdominal and pleural effusion | 0 |  | Anemia, thrombocytopenia | Tacrolimus, methylprednisolone | D34 | Hydroperitoneum 5.04*10^3^ | 252 | 9 | D34, D40, D47, D54 |
| 8 | WAS | D151 | Fever, lymphadenopathy, convulsion, hepatomegaly | 6 | submandibular and cervical lymph nodes | Anemia, leukopenia, thrombocytopenia | Tacrolimus, prednisone | D151 | Cerebrospinal fluid 1.34*10^5^ |  | 35.19 | D144, D151, D158, D165 |
| 9 | thalassemia | D23 | Fever, hepatosplenomegaly, convulsion | 2 |  | Anemia, thrombocytopenia | Cyclosporine, methylprednisolone, mycophenolate mofetil | D23 | Cerebrospinal fluid 5.31*10^3^ |  | 4.54 |  |
| 10 | thalassemia | D192 | Fever, hepatosplenomegaly, convulsion | 3 | Posterior to superior vena cava, mediastinal lymph nodes | Anemia, leukopenia, thrombocytopenia | Tacrolimus | D192 | Cerebrospinal fluid 6.9*10^2^ | 56886 |  | D192 |
| 11 | CN | D56 | Fever, lymphadenopathy, convulsion | 3 | cervical, axillary, and inguinal lymph nodes | Anemia | Tacrolimus, prednisone | D53 | Cerebrospinal fluid negative |  | 32.18 | D56, D63, D70 |
| 12 | thalassemia | D54 | Fever, hepatosplenomegaly, lymphadenopathy, convulsion | 17 | Hilar, left submandibular and cervical lymph nodes | Anemia, leukopenia, thrombocytopenia | Cyclosporine | D54 | Cerebrospinal fluid negative (but EBV-IgM positive) | >7500 | 41.53 | D55, D62, D69, D76 |

Table 2 Clinical information of four deceased patients with PTLD

|  | Patient 1 | Patient 2 | Patient 3 | Patient 4 |
| --- | --- | --- | --- | --- |
| Sex | Male | Female | Male | Female |
| Diagnosis | WAS | SCID | XLP | AA |
| Age (years) | 0.8 | 2.4 | 7.9 | 4.2 |
| Stem cell source | PB | PB | PB | BM |
| Donor type | Unrelated | Related (non-sibling) | Unrelated | Related (non-sibling) |
| HLA matching | Mismatched | Mismatched | Matched | Mismatched |
| Conditioning regimen | BU + CY + ATG | CY + FLU + ATG | BU + CY + FLU + ATG | BU + CY + FLU + ATG |
| GVHD prevention method | CsA + MMF | MTX + MMF + FK506 | CsA + MMF | CsA + MMF |
| Acute GVHD | Grade II | Grade IV | Grade III | Grade IV |
| Time of EBV reactivation (days) | 21 | 23 | 30 | 29 |
| Highest EBV-DNA (copies/ml) | 8.72 × 10^7^ | 6.27 × 10^7^ | 2.26 × 10^5^ | 2.51 × 10^7^ |
| Time of diagnosis of PTLD (days) | 272 | 162 | 45 | 78 |
| With EBV-HLH | No | Yes | Yes | No |
| Application time of rituximab (days) | 281 | 172,179,186 | 45,52,59 | 78,85 |
| Time of death (days) | 286 | 193 | 67 | 93 |
| Cause of death | Septic shock | Intestinal GVHD | Immuno pancytopenia | Multiple organ failure caused by infection |
